# A do-it-yourself water quality sensor network to elucidate contaminant signatures and improve land management advice

**DOI:** 10.1038/s41598-026-43915-9

**Published:** 2026-03-16

**Authors:** James E. Dare, Deniz Özkundakci, Richard W. McDowell

**Affiliations:** 1 School of Science, The University of Waikato, Hamilton, New Zealand; 2 Bay of Plenty Regional Council, Tauranga, New Zealand; 3 AquaWatch Solutions, Auckland, New Zealand; 4 Faculty of Agriculture and Life Sciences, Lincoln University, Lincoln, New Zealand; 5 AgResearch, Lincoln Science Centre, Lincoln, New Zealand

**Keywords:** Ecology, Ecology, Environmental sciences, Hydrology, Ocean sciences

## Abstract

Quantifying contaminant loads to estuaries is essential for setting effective limits on resource use and safeguarding ecological values. Traditional monitoring programmes often rely on infrequent sampling, which can substantially underestimate loads and obscure key transport processes. While commercial high-frequency sampling stations are prohibitively expensive, open-source, do-it-yourself technologies now offer affordable alternatives for continuous monitoring. Here, we deployed ten low-cost, in situ monitoring stations equipped with research-grade sensors across an intensively farmed catchment draining to a sensitive estuarine environment in the Bay of Plenty, New Zealand. Using artificial neural network models trained on concurrent grab samples, we converted sensor measurements into reliable 15-min estimates of contaminant concentrations. High-frequency load calculations revealed nitrogen, phosphorus, and sediment exports to be 6–87% greater than estimates derived from traditional monthly sampling. Moreover, time-series outputs uncovered distinct sub-catchment contaminant mobilisation and transport dynamics that would otherwise remain undetected. These findings demonstrate that open-source, high-frequency monitoring can substantially improve contaminant load quantification, provide new insights into catchment processes, and inform the development of land management and policy strategies that reflect the unique spatio-temporal patterns of contaminant export.

**Supplementary Information:**

The online version contains supplementary material available at 10.1038/s41598-026-43915-9.

## Introduction

Agricultural intensification and urbanisation has led to widespread degradation of freshwater quality globally, with increased contaminant losses and declining water quality in many rivers, lakes, and estuaries^1–4^. Effective management of these systems requires accurate monitoring of water quality, which is essential for understanding long-term changes, estimating contaminant loads, and identifying the drivers of ecological decline in affected catchments^5^. In many regions, local authorities use a combination of policy tools, regulatory processes, and on-the-ground action to manage freshwater resources. However, traditional water quality monitoring programmes with monthly or greater sampling frequency, often fail to capture rapid hydrochemical processes and changes that occur on shorter time scales^6,7^. Kirchner^6^ likens this to ‘trying to understand a symphony by listening to only one note every minute or two’. However, this symphony occurs across an entire catchment, where discrete moments in space and time are known to contribute disproportionately relative to background conditions. While datasets from monthly sampling are suitable for some analytical purposes such as trend analysis^8^ or the calculation of crude contaminant loads, the lack of temporal resolution limits the ability to identify key processes where contaminant mobilisation is most acute, resulting in an incomplete understanding of water quality dynamics^6^.

Recent advances in the concept of ‘hot-spots and hot-moments’ (HSHM) have highlighted the importance of temporal and spatial variability in biogeochemical processes and contaminant mobilisation^9,10^. HSHM refers to specific locations and times periods within a catchment where biogeochemical reactions and contaminant fluxes are disproportionately high. Understanding these phenomena is critical for improving water quality management, as areas with elevated biogeochemical process rates (e.g., wetlands, or riparian zones) can be protected, enhanced, or created to limit the extent of problems such as eutrophication^10^. Resource managers also need to understand HSHM as sources of contaminants to be able to prioritise the location of management interventions, as well as understanding the relative importance of ‘hot-moments’^10^.

Traditional monthly water quality monitoring does not adequately characterise ‘hot-moments’ which can lead to underestimation of contaminant loads due to the disproportionate contribution of short-lived storm events^11^. For example, Abell et al.^12^ showed that annual load estimates of total nitrogen (TN) and total phosphorus (TP) in two New Zealand streams were 19% and 40% less when estimated via monthly samples versus samples supplemented with targeted storm monitoring. Furthermore, McDowell et al.^13^ found that monthly sampling resulted in a ~ 30% error in the estimation of TP and nitrate-nitrogen loads compared to sampling every 30 minutes.

Knowledge of HSHM is particularly important for implementing environmental policy in New Zealand (the focus areas of this study) within the context of New Zealand’s National Policy Statement for Freshwater Management^14^. This policy mandates regional authorities to set contaminant limits to prevent further water quality decline, and to achieve outcomes desired by their local community. However, underestimation of contaminant loads from traditional monthly sampling could lead environmental managers to make incorrect conclusions about the severity or location of contaminant issues, leading to weak or ineffective management actions that will never achieve the intended objective^13^. By enhancing monitoring efforts to capture HSHM, environmental managers could more accurately quantify contaminant loads and develop the most appropriate management strategy to match contaminant mobilisation nuances observed in discrete areas of the catchment^15^.

The expansion of in-situ water quality sensors provides an opportunity to capture HSHM at discrete sites within a monitoring network. However, commercially available monitoring stations are expensive which means that regional authorities are often limited to a small number of sites in large river network catchments^16,17^. An emerging solution comes via the ‘open-source movement’, which aims to make equipment and software available for modification with a comparably low-cost barrier to entry^18^. This has led to the development of ‘do-it-yourself’ (DIY) environmental monitoring options that are able to control an array of research grade sensors for a fraction of the price of commercial monitoring stations. One such option is the EnviroDIY Mayfly board, which has been designed for low-cost, solar-powered, wireless applications in environmental science^16^. Users can programme the board themselves using a range of different sensor-specific code templates written within the Arduino interactive development environment (IDE) and shared via an active GitHub community, providing similar functionality and reliability to commercially available systems.

Environmental practitioners often use in-situ sensor data to develop a relationship with hydrochemical or physical parameters measured via lab-samples, in a process referred to as a surrogate relationship^19^. Surrogate relationships provide a practical, cost-effective method to understand high-frequency, hydrochemical dynamics with more accuracy than can be achieved using discrete water quality samples alone^19,20^. Surrogate relationships have evolved from stepwise regression models to more computationally intensive machine learning models such as random forest or artificial neural networks (ANN)^21,22^. The use of ANN methods have been a popular choice for water quality studies due to their robustness, ability to resolve nonlinear problems, absence of statistical assumptions, and suitability to smaller datasets compared with other machine learning techniques^21,23,24^. Examples of ANN applications in the field of water quality research include: the prediction of water quality index scores from hydrochemical measurements in South African river basins^25^; estimation of microcystin concentrations in the Binhu river network of China^26^; prediction of TN and TP concentrations in US lakes from in-situ pH, conductivity, and turbidity measurements^27^; the prediction of turbidity in the Nalón river basin (Spain) from in-situ sensor measurements^28^; and detection of spills and discharges into the Potomac River Basin in Virginia, USA^29^.

The main objectives of this study are twofold:

1. To develop a low-cost monitoring network using DIY water quality stations, in combination with ANN-based surrogate models, to elucidate fine-scale catchment dynamics and enhance the information obtained from a traditional water quality monitoring network.
2. To use the high-frequency data generated by this network to identify contaminant HSHM, and to demonstrate how these insights can be translated into improved, catchment-specific resource management recommendations.

It is hypothesised that the enhanced monitoring network will provide a pragmatic means of resolving contaminant HSHM and improving the accuracy of contaminant load estimation relative to conventional monitoring approaches.

## Methods

### Study area

**Fig. 1:**
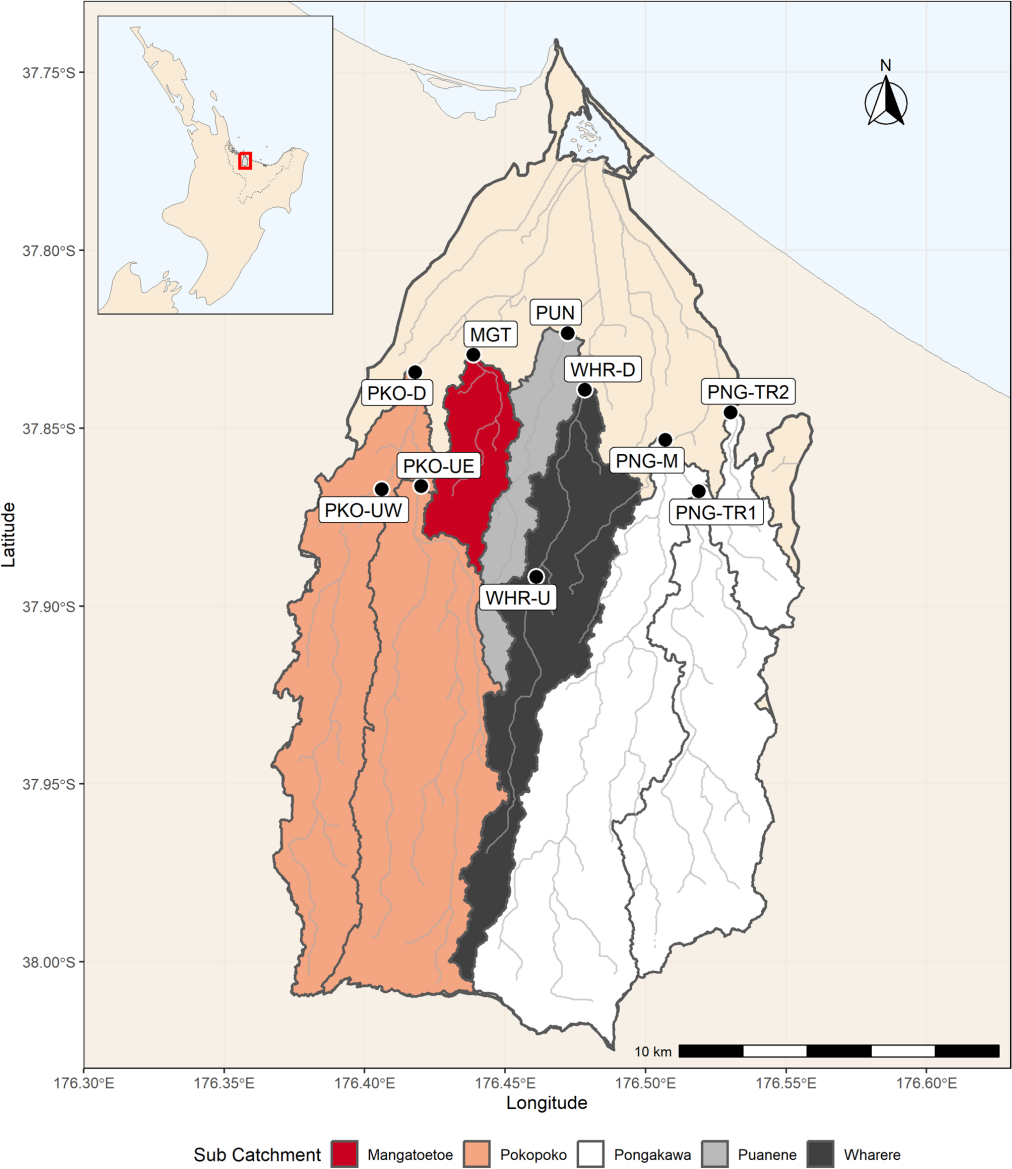
The Waihi Estuary Catchment in the Bay of Plenty region of New Zealand. The ten study sites are shown as black points and labelled using site codes. The five monitored sub-catchments are coloured according to the legend, while the unmonitored lower catchment is shown using the default shading. Site PUN is also a permanent hydrological monitoring site maintained by the Bay of Plenty Regional Council. Maps were generated by the authors in R v4.5.1 using the sf package (https://cran.r-project.org/).

This study was carried out in the Waihi Estuary catchment, near the township of Pukehina, in the Bay of Plenty region of New Zealand (Fig. 1). The Waihi Estuary catchment is predominantly agricultural and covers approximately 34,256 hectares at the north-western edge of the Taupo Volcanic Zone^30^. Soil types are predominantly well-drained pumice soils overlying ignimbrite in the upper and mid catchment, transitioning to poorly drained gley soils overlying swamp deposits near the estuary^31,32^. Major land uses include dairy farming (35%), exotic forestry (20%, mainly *Pinus radiata*), sheep and beef farming (14%), and orchards (7%, mainly kiwi fruit). The catchment has undergone extensive hydraulic modification, particularly in lowland areas, where wetlands fringing Waihi Estuary have been drained, and riverine inputs have been channalised, to make way for agricultural intensification.

The catchment drains into Waihi Estuary, a shallow, eutrophic, intertidal estuary that is currently experiencing nuisance blooms of macroalgae due to excess nutrient and fine sediment loading from the contributing catchment^33^. A catchment modelling study carried out in 2014 estimated that TN, TP, and total suspended solids (TSS) loads are currently 524%, 124%, and 232% of their natural state^33^. Furthermore, it has been estimated that TN and TSS loads need to be reduced by approximately 40% and 65%, respectively to achieve a state that supports biodiversity, ecological functioning, and provides for cultural and community values^34^. Estimated TP load reductions are more complex; work by Atkinson and Smith^35^ implies that an N: P ratio of 30:1 is an ideal standard for assessing nutrient limitation in estuaries, which translates to a target of 18.2 t yr^− 1^, or an approximate TP reduction of 30% from the 2014 load^33^. However, ‘natural state’ modelling implies that the natural N: P ratio was historically around 6.4:1 due to natural, volcanic, sources of P within the catchment, which means that the ideal ratio of 30:1 may be unrealistic^33^. Total phosphorus remains a focus in this study given that opportunities to manage anthropogenic sources are still abundant and important for managers to understand.

### Traditional water quality monitoring investigation

 Bay of Plenty Regional Council (BOPRC), the regional authority that is responsible for managing water resources in the Bay of Plenty, established a routine water quality monitoring network in the Waihi Estuary catchment in 2021. This traditional monitoring network is part of BOPRC’s ‘focus catchment programme’, which aims to identify high-risk areas within a deteriorating catchment by increasing the spatial resolution of water quality monitoring sites. Monthly water quality samples were collected by trained professionals at 10 representative sites throughout the catchment (Fig. 1), and analysed for a suite of constituents, including TN, TP, and TSS. This dataset was used as the foundation for the current study. Monitoring sites are referred to using short site codes throughout the manuscript; full site names, locations, and code mappings are provided in the supplementary information (Table S1).

### Continuous monitoring

For the current study, each of the 10 sites in the BOPRC investigation was supplemented with a ‘Mayfly’ sensor station to better characterise riverine conditions between sampling dates. Sensor stations were built using an EnviroDIY Mayfly data logger board, which is an arduino-based microcontroller that has been developed for low-cost environmental research[36]. Each Mayfly board was housed within a 2.4 L Pelican^TM^ box and attached to a 1.5 m pole on the riverbank. Sensor stations were equipped with a 3.7 V 3800mAh LiPo battery pack and a 6 V 3.5 W Voltaic solar panel providing an indefinite power source.

Two factory calibrated water quality sensors were attached to each sensor station: a HYDROS-21 conductivity, temperature, and depth sensor (CTD) from METER Group, and a Y11-A nephelometer with wiper from Yosemite Technologies. This sensor combination is recommended for detecting changes in dissolved contaminants (e.g., nitrate-nitrite-N [NNN], dissolved reactive P [DRP]) as well as particulate contaminants (e.g., TN, TP, TSS)^16^, while being price competitive, reliable, and compatible with the Mayfly system. CTD and turbidity data for each site were captured at 15-minute intervals and stored on a removable SD memory card.

Sensors were maintained via monthly site visits which involved removal of debris, thorough cleaning, and repairing of any damaged wipers. Data was downloaded at six monthly intervals and uploaded to a water management database. This database was designed for hydrological and water quality datasets and contains numerous in-built tools that enable data QA/QC processes. The four continuous data records from each monitoring site were assessed by a qualified environmental data specialist following upload to the database system. The data specialist was tasked with removing erroneous spikes, cross checking abnormalities with field notes, comparing data records to discrete field validation measurements, and adjusting datasets where appropriate (e.g., where sensor drift had occurred).

### Storm event sampling

Stormflow sampling was used to capture contaminant dynamics during high-flow periods, which are known to contribute disproportionately to annual nutrient and sediment loads. Stormflow samples were collected using one of two available Teledyne ISCO 6715 autosamplers which were deployed opportunistically in response to incoming weather events. The intake hose of each autosampler was positioned next to the sensor station at each site to ensure that samples were representative of the conditions recorded by the sensors. Autosamplers were programmed to collect samples at a set time interval, commencing in pre-event conditions and, ideally, finishing when the hydrograph returned to baseflow. This generally equated to one sample every 90 min throughout an event.

A total of 21 storm events were captured across all 10 monitoring sites, with each site being sampled during at least two weather events. Storm event sampling lasted an average of 35 h and represented rainfall accumulations of 27 mm to 116 mm.

### Laboratory analysis

All hydrochemical analyses for the routine monitoring programme, as well as additional storm-event samples, were carried out by the BOPRC water quality laboratory using IANZ-accredited methodologies. Nitrogen and phosphorus concentrations used in this study were analysed as total fractions (TN and TP) using persulphate digestion (APHA 4500-P J^37^) and flow injection analysis. While additional nutrient fractions were analysed as part of the broader monitoring programme, only total nutrient concentrations were used for surrogate modelling and load estimation in this study. Total suspended solids (TSS) were measured using a standard glass fibre filter method (APHA 2540 D^38^).

### Discharge measurements

High-frequency discharge measurements were required at each site to allow for calculation of instantaneous loads. Puanene (PUN) met this requirement as it was a permanent hydrological monitoring site maintained by BOPRC, however discharge records at the other nine sites required development. This process involved manually measuring stream discharge on at least five dates over the latter half of 2022, using either a SonTek FlowTracker2 Acoustic Doppler Velocimeter, a SonTek-RS5 ADCP, or a SonTek M9. These data were combined with sensor water level readings and analysed by qualified BOPRC hydrologists to develop water level-discharge rating curves for each site. Four of the nine rating curves were unreliable due to inconsistent cross-sectional areas. This was addressed at three of the four sites by developing linear relationships between the discharge measurements and continuous discharge measurements from PUN. Discharge at Pongakawa mainstem (PNG-M) was estimated through a multi parameter model that incorporated discharge from a permanent hydrology site in a neighbouring catchment (Waitahanui at Otamarakau Valley Rd, approximately 1 km away from the study catchment), water level from a nearby lake (Lake Rotoma, approximately 7.5 km away from the study catchment), and calendar year. Discharge records were summarised to 15-minute intervals to match the frequency of data from each Mayfly station. Details for each flow relationship are shown in Table 1.

**Table 1:** Discharge relationships developed for each of the ten monitoring sites.

Site code	Discharge model type	Predictor variables	Performance
MGT	Linear relationship	Stream discharge at PUN	*P* < 0.01 R² = 0.84
PKO-UE	Rating curve	Water level	*P* < 0.05 R² = 0.73
PKO-UW	Rating curve	Water level	*P* < 0.001 R² = 0.99
PKO-D	Linear relationship	Stream discharge at PUN	*P* < 0.001 R² = 0.93
PNG-M	Artificial Neural Network	Stream discharge at Waitahanui at Otamarakau Valley Rd Lake level at Rotoma at Whangaroa Calendar year	RMSE = 0.023 R² = 0.86 NSE = 0.84 PBIAS = −0.2
PNG-TR1	Rating curve	Water level	*P* < 0.05 R² = 0.96
PNG-TR2	Linear relationship	Stream discharge at PUN	*P* < 0.01 R² = 0.81
PUN	Rated hydrological site	Water level	*P* < 0.001 R² = 0.99
WHR-U	Rating curve	Water level	*P* < 0.001 R² = 0.95
WHR-D	Rating curve	Water level	*P* < 0.05 R² = 0.79

### Data analysis

All statistical, modelling, and geospatial analyses were conducted in R v4.5.1^39^.

TN, TP, and TSS concentrations from BOPRC’s routine sampling programme, and the targeted storm event monitoring programme, were extracted for each site and combined with mean-hourly or mean half-hourly sensor values. Missing sensor values were imputed by linear interpolation for gaps smaller than four hours using the na.approx function in the zoo^40^ package, and laboratory measurements that were below the 5th or beyond the 95th percentile were treated as outliers and removed from the dataset. Final datasets ranged from 64 to 101 observations, depending on the site and parameter.

Datasets for each site-contaminant combination were used to develop real-time surrogate contaminant concentration records using feed-forward, back-propagation artificial neural network (ANN) models^27^. In total, separate models were developed for TN, TP, and TSS at each of the ten monitoring sites (30 models). Candidate predictor variables were derived from in-situ sensor measurements, including conductivity, temperature, modelled discharge, and turbidity, with either TN, TP, or TSS concentration used as the response variable.

The general modelling procedure was consistent across all site-contaminant combinations, although the final predictor set varied depending on data availability and observed relationships. Datasets were first visually explored using the ggplot2^41^ package to assess relationships between sensor measurements and response variables, and to inform the selection of predictor variables and any statistical transformations. Each dataset was then split into training (70%) and testing (30%) subsets, with stratification by event status to ensure that event and non-event samples were proportionally represented in both subsets.

ANN models were developed within the tidymodels^42^ framework using the brulee^43^ engine. Models consisted of single-hidden-layer multilayer perceptrons configured for regression. Model training was performed using stratified ten-fold cross-validation applied to the training dataset, and a grid search was used to tune four hyperparameters: number of training epochs, number of hidden units, weight decay penalty, and learning rate. The optimal hyperparameter combination was selected by minimising cross-validated root mean squared error (RMSE).

 Finalised models were evaluated against the held-out test dataset, and performance was assessed using the coefficient of determination (R²), Nash–Sutcliffe efficiency (NSE), percent bias (PBIAS), and RMSE, alongside visual inspection of predicted versus observed concentrations. Models with NSE < 0.5, or exhibiting systematic bias or non-physical behaviour evident in diagnostic plots, were deemed unacceptable and excluded from further analysis. Accepted models were subsequently applied to continuous 15-minute sensor records to generate high-frequency surrogate concentrations of TN, TP, and TSS. Upper and lower 90% prediction intervals were estimated for each modelled datapoint using split conformal inference implemented via the probably^44^ package. The architecture and tuned hyperparameters of retained ANN models are summarised by contaminant and across the network in the supplementary information (Table S2).

### Flow partitioning

Modelled discharge for each site was separated into baseflow and event-flow using a Lyne Hollick recursive baseflow filter^45^ within the grwat^46^ package. This baseflow separation method is widely accepted to be simple, robust and repeatable, was the preferred method for the New Zealand baseflow index characterisation project, and is well suited for streams where a substantial proportion of flow originates from groundwater^47^. A filter parameter of 0.99 was selected for all sites by comparing the saturated hydraulic connectivity of volcanic soils in Alavani and Tomer^48^ with optimal filter parameter values in Li et al.^49^.

### Contaminant load calculation

Load calculations were only performed for site-parameter combinations where surrogate model performance met predefined acceptance criteria (NSE ≥ 0.5 and no evidence of systematic bias or non-physical behaviour in diagnostic plots). Contaminant loads for TN, TP, and TSS were calculated for each site according to Eq. 1, derived from the numeric integration method in Meals et al.^11^, where c_i_ is the ith modelled concentration value, q_i_ is the corresponding modelled flow, and t_i_ is the time interval between measurements (fifteen minutes). Sensor data gaps greater than four hours were removed from the dataset to avoid bias.

1 $$MeanAnnualLoad=\left(\frac{{\sum}_{i=1}^{n}{c}_{i}{q}_{i}{t}_{i}}{{\sum}_{i=1}^{n}{t}_{i}}\right)\cdot secondsperyear$$

The same equation was also applied to upper and lower 90% prediction interval datasets. Mean annual baseflow loads were calculated in the same way with the exception of using scaled flow output from the recursive baseflow filter.

### Comparison with traditional load estimation

Mean annual load estimates calculated from ANN models were compared to estimates calculated from monthly samples for each site-contaminant combination. Datasets for the traditional method required removal of autosampler and opportunistic samples, leaving 24–29 discrete monthly samples at each site.

Symmetric 90th percentile confidence intervals were calculated for the traditional method using Eq. 2^8^, where $\mu$ is the true population mean, ${t}_{\left(\frac{a}{2},n-1\right)}$ and ${t}_{\left(1-\frac{a}{2},n-1\right)}$ are critical t values with $a=0.95$, $n$ = sample size, $\bar{x}$ = mean load estimate, and $s$ = the standard deviation of $\bar{x}$.

2 $${{\bar{x}+{t}_{\left(\frac{a}{2},n-1\right)}}_{}\cdot\sqrt{\frac{{s}^{2}}{n}}\le\mu\le\bar{x}+{t}_{\left(1-\frac{a}{2},n-1\right)}\cdot\sqrt{\frac{{s}^{2}}{n}}}_{}$$

### Spatial analysis

Spatial analysis was carried out using the sf^50^ package. Contributing sub-catchments for each monitoring site were delineated by combining upstream watersheds from the River Environment Classification (REC) digital river network^51^. This information was used to calculate the annual areal load for each sub-catchment (kg ha^− 1^ yr^− 1^).

## Results

### Routine monitoring overview

 The number of routine water quality samples collected at each site between November 2021 and June 2024 ranged from 24 to 29. This did not include targeted storm samples or opportunistic samples and therefore represents ‘traditional monitoring’ initiated by BOPRC. Summary statistics for TN, TP, and TSS for each network site are available in the supplementary information (Table S3).

Median TN concentrations ranged from 0.99 mg L^−1^ at Pokopoko upper west (PKO-UW) to 2.37 mg L^−1^ at Wharere downstream (WHR-D). Maximum TN concentrations were highest at Puanene (PUN; 3.55 mg L^−1^) and lowest at Pongakawa mainstem (PNG-M). PUN and Mangatoetoe (MGT) exhibited the greatest variability in TN (standard deviation ≈ 0.60 mg L^−1^), compared with a mean site standard deviation of 0.32 mg L^−1^. Median TP concentrations ranged from 0.091 mg L^−1^ at PKO-UW to 0.133 mg L^−1^ at PUN and Pongakawa tributary 1 (PNG-TR1). PUN exhibited a maximum TP concentration 51 times greater than its median value (6.73 mg L^−1^), compared with an average factor of 4.4 across the remaining sites. Median TSS concentrations ranged from 3.8 mg L^−1^ at Wharere upstream (WHR-U) to 55.9 mg L^−1^ at PKO-UW, while maximum TSS concentrations ranged from 89.0 mg L^−1^ at WHR-U to 1940.0 mg L^−1^ at PKO-UW. Five sites recorded maximum TSS concentrations more than 30 times their median values: PKO-UW, Pokopoko upper east (PKO-UE), Pokopoko downstream (PKO-D), PNG-TR1, and Pongakawa tributary 2 (PNG-TR2). Three of these sites were located within the Pokopoko sub-catchment, while PNG-TR1 and PNG-TR2 were located within the Pongakawa sub-catchment.

### Storm event monitoring

**Fig. 2:**
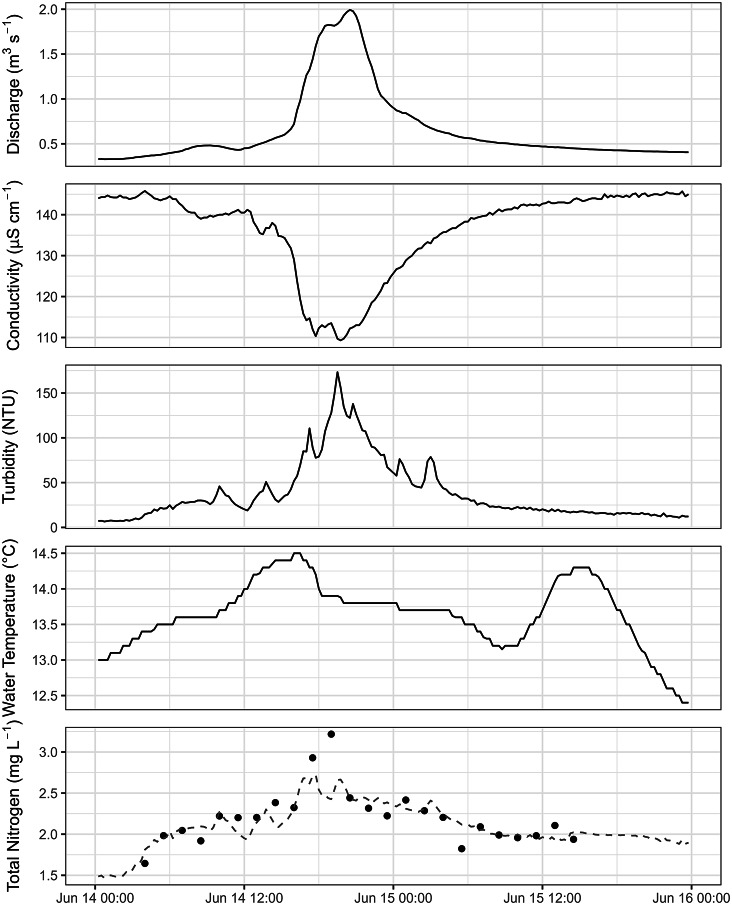
Data collected during a storm event that occurred on 14 June 2024 at MGT. Panels show modelled discharge (top panel), in-situ conductivity (second panel), turbidity (third panel), and water temperature (fourth panel), above discrete lab-measured TN concentrations (bottom panel). The dashed line on the bottom panel shows the predicted TN record for this event derived from the surrogate relationship.

An example of targeted storm event monitoring at MGT is shown in Fig. 2. Discharge peaked at 2.02 m^3^ s^− 1^ at 20:30 on June 14 2024, reflected by a drop in specific conductivity from a pre-event baseline of approximately 130 µS cm^− 1^ to a minimum of 60 µS cm^− 1^. Turbidity increased with discharge to approximately 175 NTU, before returning to pre-event levels as the discharge receded. Water temperature was less affected by the storm event, and more controlled by diurnal patterns, increasing to a peak of 14.5 °C in the afternoon before reducing into the evening.

The first autosampler sample was collected at 04:00 on June 14 2024, continuing until 24 samples had been collected at 14:30 on June 15 2024. The results for TN are displayed in the bottom panel of Fig. 2 and show a pre-event concentration of 1.64 mg L^− 1^, increasing to a peak of 3.21 mg L^− 1^ which occurred slightly before the discharge peak, reducing to a post-event concentration of 1.93 mg L^− 1^.

The dashed line in the bottom panel represents modelled TN concentrations, where discharge, conductivity, and turbidity were used as predictor variables. The model was able to accurately represent measured TN concentrations throughout this event, with the exception of the two highest values that occurred just before the discharge peak, and one value that occurred just before 06:00 on June 15.

### Surrogate relationships

The overall performance of ANN models was good, with 53% of models across all parameters having a Nash-Sutcliffe efficiency (NSE) value > 0.8, and 76% of models having a NSE > 0.7. TSS was the best performing contaminant model across all sites (median NSE = 0.90), followed by TP (median NSE = 0.80) and TN (median NSE = 0.75) (Table 2).

Model performance was generally poorer at sites that were influenced by groundwater, such as WHR-U (mean NSE = −0.34 across all parameters), and PNG-M (mean NSE = 0.77). All contaminant models for WHR-U and PNG-TR2 were removed from further analysis due to poor diagnostic performance (NSE < 0.5). As a result, these sites were excluded from subsequent calculations of contaminant loads, yields, and baseflow contributions, and are explicitly identified as unmodelled and excluded from all relevant figures.

**Table 2:** Model performance for each site and parameter across the study period (November 2021 - June 2024). The coefficient of determination (R^2^), Nash-Sutcliffe efficiency (NSE), percent bias (PBIAS), and root mean squared error (RMSE) values are shown as indicators of model performance. ‘Storm Events’ shows the number of individual storm events that were included in the model dataset at each site, and ‘Storm Sample Composition’ provides the percentage of storm event samples relative to the entire dataset for that parameter.

Site code	Parameter	Storm events	Storm sample composition	*R* ^2^	NSE	PBIAS	RMSE
MGT	TN	2	55%	0.74	0.72	−2.7	0.04
	TP	2	55%	0.81	0.79	0.5	0.10
	TSS	1	44%	0.92	0.92	2.0	0.18
PKO-UE	TN	2	62%	0.88	0.83	−7.9	0.09
	TP	2	62%	0.81	0.80	3.5	0.17
	TSS	2	67%	0.95	0.94	1.5	0.14
PKO-UW	TN	2	54%	0.80	0.79	7.5	0.06
	TP	2	54%	0.87	0.85	−5.2	0.11
	TSS	2	58%	0.87	0.85	−1.5	0.17
PKO-D	TN	2	59%	0.86	0.83	3.0	0.04
	TP	2	59%	0.83	0.82	0.2	0.18
	TSS	2	67%	0.98	0.98	−0.1	0.12
PNG-M	TN	3	56%	0.68	0.66	2.1	0.02
	TP	3	60%	0.71	0.71	−0.3	0.06
	TSS	1	44%	0.97	0.94	−3.7	0.10
PNG-TR1	TN	2	56%	0.66	0.65	−1.1	0.03
	TP	2	56%	0.76	0.74	0.8	0.94
	TSS	2	63%	0.87	0.79	1.6	0.10
PNG-TR2	TN^2^	2	42%	0.55	0.45	2.4	0.06
	TP^2^	2	42%	0.91	0.82	4.1	0.07
	TSS^2^	1	45%	0.84	0.84	−6.3	0.15
PUN	TN	2	56%	0.84	0.83	−0.7	0.07
	TP	2	56%	0.71	0.59	−2.0	0.23
	TSS	1	43%	0.95	0.87	−7.8	0.22
WHR-U	TN^1^	2	46%	0.36	−0.23	−0.7	0.03
	TP^1^	2	46%	0.01	−0.98	6.7	0.22
	TSS^1^	2	50%	0.20	0.19	−2.7	0.28
WHR-D	TN^3^	2	54%	0.79	0.78	−0.9	0.04
	TP	2	54%	0.93	0.90	4.1	0.09
	TSS	2	59%	0.97	0.96	2.7	0.10

^1^Model was deemed unacceptable because NSE < 0.5.

^2^Model was deemed unacceptable based on visual inspection.

^3^Model was deemed acceptable but is considered to be of lower quality after visual inspection, despite acceptable model performance indicators.

**Fig. 3:**
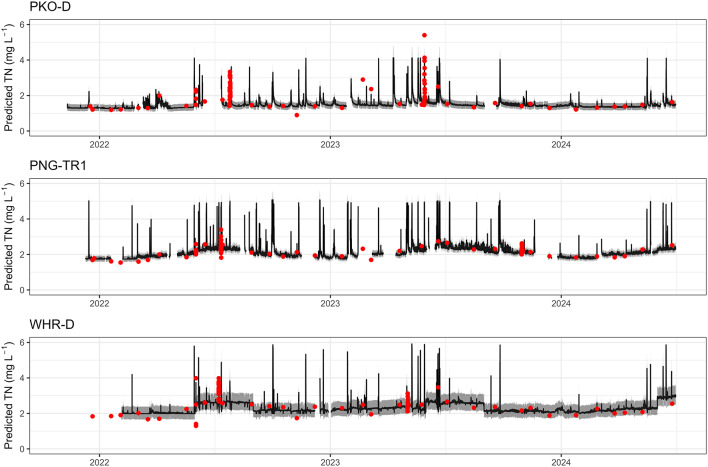
Examples of modelled TN concentrations for three of the ten network sites. The black lines represent predicted TN, the grey shaded bands represent the upper and lower 90% prediction intervals, and the red points represent discrete laboratory sample results.

Figure 3 provides examples of modelled TN for three of the ten network sites. The shaded bands represent upper and lower 90% prediction intervals, and the red points represent observed TN concentrations from laboratory samples. All three models provide a good fit between predicted and observed data, with NSE values of 0.83, 0.65, and 0.78 (top to bottom). However, the bottom model was considered lower quality than the others due to data steps and reduced certainty (wider prediction intervals), despite having a NSE value of 0.78. These examples show the importance of visually inspecting model output in addition to statistical output.

### Total load export

The summed mean annual load export from the most downstream site on each river branch (henceforth ‘export sites’) was 550 t TN yr^− 1^, 56 t TP yr^− 1^, and 36,300 t TSS yr^− 1^.

**Fig. 4:**
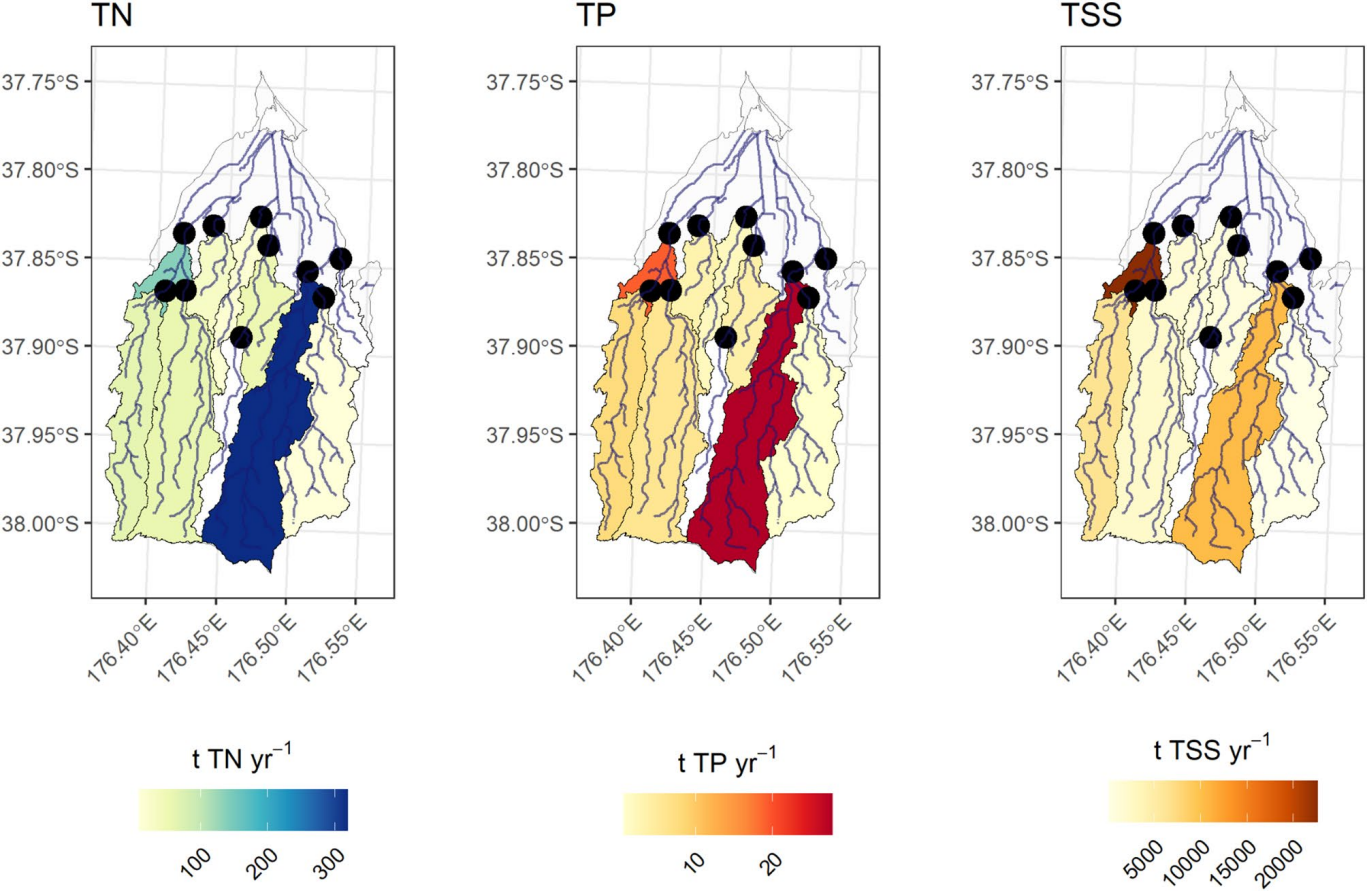
Total annual loads of TN, TP, TSS at the ten monitoring sites in the Waihi Estuary catchment for data collected between November 2021 and June 2024. Areas in white represent poor quality contaminant models that were removed from the analysis or unmonitored sections of the lower catchment. Maps were generated by the authors in R v4.5.1 using the sf package (https://cran.r-project.org/).

PNG-M had the highest mean TN and TP load export, contributing 58% (319 t yr^− 1^) of the total TN load, and 49% (27.8 t yr^− 1^) of the total TP load exported (Fig. 4). PKO-D contributed 25% of the TN (136 t yr^− 1^) and 34% of the TP (19.1 t yr^− 1^) load. TN and TP export at PKO-D was 4% and 10% greater than the combined loads from upstream sites (PKO-UW, PKO-UE) for TN and TP, respectively. The combined load export from other sites within the catchment made up approximately 17% of the total load export for both TN and TP.

Sediment load export was dominated by the Pokopoko sub-catchment, with PKO-D exporting 62% (22,570 t yr^− 1^) of the total annual network load. The combined export from the two upstream branches of the Pokopoko Stream equated to 23% of the total network load, implying that 39% of the annual network load is generated in the small area between these sites. PNG-M contributed 28% (10,313 t yr^− 1^), and all other river branches contributed a combined annual load ~ 10%.

### Contaminant yields

**Fig. 5:**
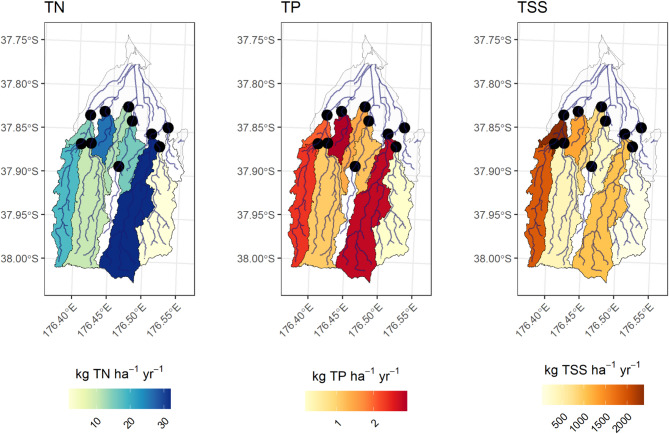
Yields of TN, TP, TSS at the ten monitoring sites in Waihi Estuary catchment for data collected between November 2021 and June 2024. Areas in white represent poor quality contaminant models that were removed from the analysis or unmonitored sections of the lower catchment. Maps were generated by the authors in R v4.5.1 using the sf package (https://cran.r-project.org/).

Total nitrogen yields were highest at PNG-M (32.0 kg ha^− 1^ yr^− 1^), followed by MGT (25.9 kg ha^− 1^ yr^− 1^) and PKO-UW (18.4 kg ha^− 1^ yr^− 1^) (Fig. 5). MGT expressed the largest TP yield (2.9 kg ha^− 1^ yr^− 1^), followed by the PNG-M (2.8 kg ha^− 1^ yr^− 1^), and the PKO-UW (2.4 kg ha^− 1^ yr^− 1^). Sediment yields were largest for PKO-D (2,385 kg ha^− 1^ yr^− 1^) and could be traced upstream to PKO-UW (1,911 kg ha^− 1^ yr^− 1^). Other significant contributors of sediment were MGT (1,284 kg ha^− 1^ yr^− 1^) and PNG-M (1,034 kg ha^− 1^ yr^− 1^).

PNG-TR1 had the lowest yield across all three contaminants.

### Baseflow load export

**Fig. 6:**
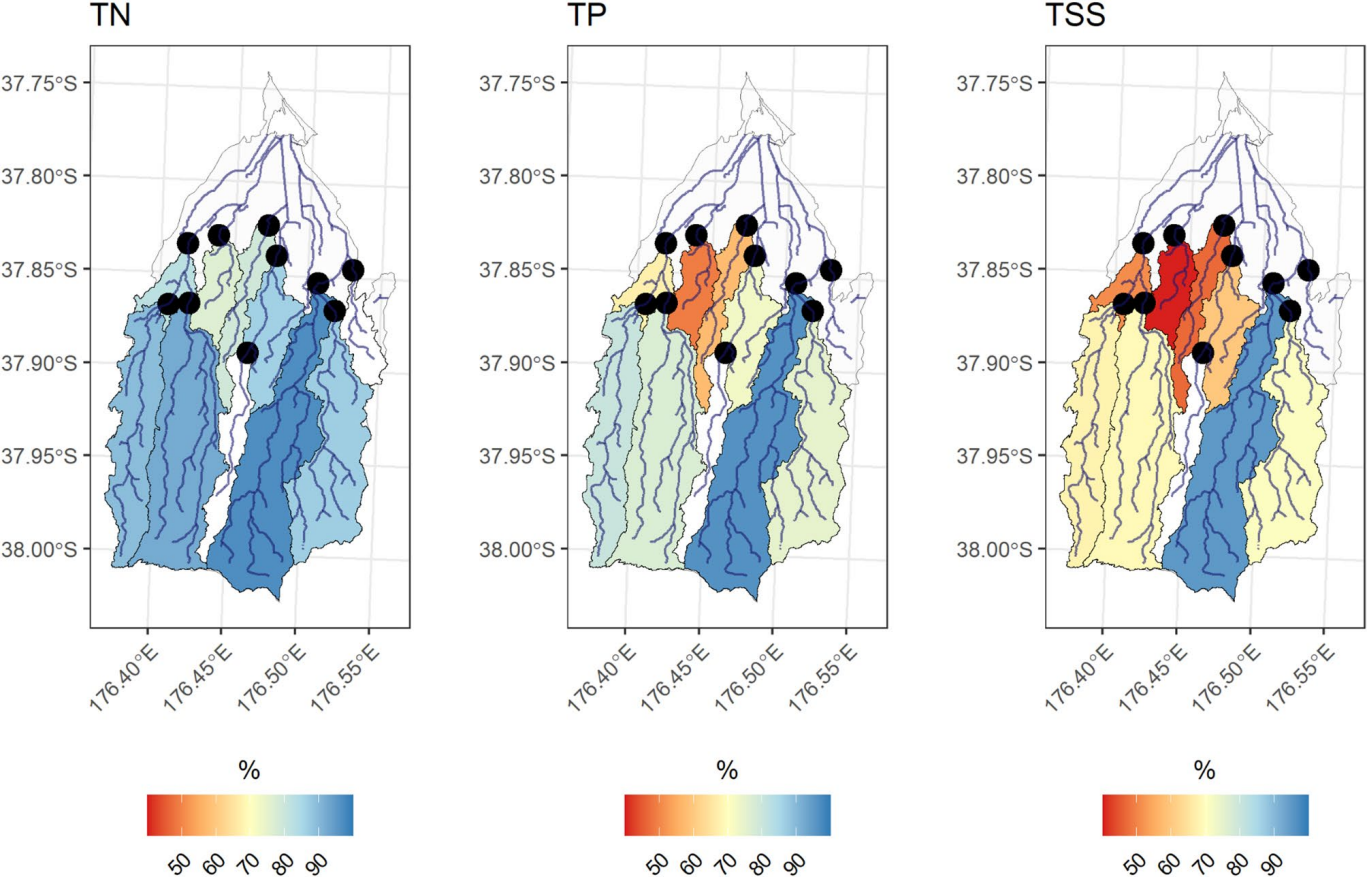
The average percentage of TN, TP, TSS load that was delivered during baseflow conditions, throughout the study period (November 2021 - June 2024). Areas in white represent poor quality contaminant models that were removed from the analysis or unmonitored sections of the lower catchment. Maps were generated by the authors in R v4.5.1 using the sf package (https://cran.r-project.org/).

The percentage of TN load exported during baseflow conditions varied from 76% to 97% (Fig. 6). The most baseflow dominant sites were PNG-M (97%), and the two upper Pokopoko catchment sites (PKO-UE = 92%; PKO-UW = 89%). The least baseflow dominant sites were in the lower catchment (MGT = 76%; PUN = 79%; PKO-D = 82%).

Baseflow TP export was more variable between sub-catchments than TN, ranging from 49% to 96%, and more controlled by non-baseflow events. Sites with the largest baseflow TP delivery were located in the Pongakawa or upper Pokopoko sub-catchments. Similar to TN, catchments closer to the estuary were more controlled by event-flow delivery (e.g., MGT = 49%; PUN = 58%).

TSS was the most variable contaminant across sites for baseflow export (40%−95%), and the most event-driven. Two sites in the Pongakawa sub-catchment (PNG-M; PNG-TR1), delivered the greatest percentage of TSS load during baseflow conditions, with 95% and 86%, respectively. Conversely, MGT and PUN delivered the highest percentage of TSS load during storm events, with baseflow delivery percentages of 40% and 47%, respectively.

### Comparison of load estimation methods

**Fig. 7:**
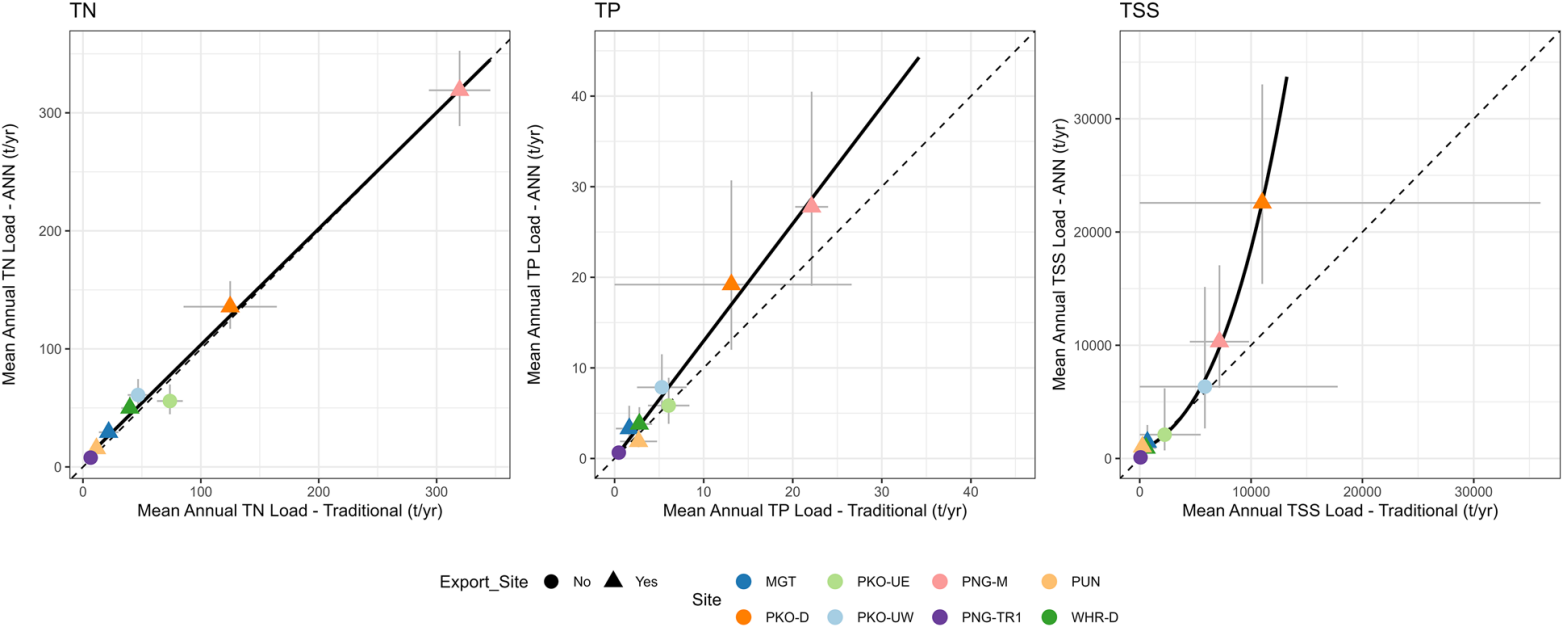
Comparison of mean annual loads for each site calculated from ANN models (y axis) and traditional monthly sampling (x axis), for TN (left), TP (middle), and TSS (right). Circles represent the most downstream sites in each sub-catchment (i.e., contributing sites). Horizontal and vertical error bars represent 90% CI’s, the diagonal dashed line shows a 1:1 ratio, and the black line shows a model of best fit.

Figure 7 shows a comparison of load estimates calculated from ANN models and discrete monthly samples (henceforth the ‘traditional’ method). TN load estimates were approximately equal between the two methods, however TP and TSS estimates diverged, particularly for sites with larger estimated loads. ANN model estimates were on average 5% (−24.3% to 36.5%), 29% (−30% to 101%), and 61% (−6% to 315%) larger than traditional estimates, for TN, TP, and TSS, respectively. Export sites had total combined loads for TN, TP and TSS that were 6%, 32%, and 85% larger for ANN model estimates than the traditional method.

The width of confidence intervals (CI’s) was contaminant dependent, with the narrowest CI’s calculated across both methods for TN (average width = 56% of estimate), and the widest CI’s calculated for TSS (average width = 284% of estimate). Confidence intervals were on average 2% (−66% to 56%), and 74% (−61% to 482%) wider for ANN models compared to the traditional method for TN and TP, and 8% (−65% to 104%) narrower for TSS.

## Discussion

### Performance and value of high-frequency surrogate monitoring

This section evaluates the performance and added information gained from high-frequency surrogate monitoring using DIY sensors and ANN models, providing context for subsequent management interpretation.

The current study is among the first to apply DIY sensor technology and surrogate modelling across multiple sites within a single catchment, for the purpose of resolving spatio-temporal contaminant mobilisation. Our results show that integrating traditional monitoring with targeted storm-event sampling and low-cost sensors yields critical understanding of catchment function, particularly the timing and magnitude of nutrient delivery during high flows.

Surrogate water quality models have been widely applied in other contexts, with reported performance broadly comparable in magnitude to our findings. For example, Harrison et al.^52^ pooled data across multiple River Thames sites using random forest models to predict TN and TP, achieving NSE values of 0.85 and 0.74 (compared with averages of 0.76 and 0.78 in this study). Shyu et al.^53^ developed surrogate models for wastewater treatment constituents, reporting R² values of 0.46–0.97 (0.84–0.97 in this study), while Holmberg et al.^54^ used ANN models in boreal streams to predict TN and TP with R² values of 0.83 and 0.78 (0.76 and 0.82 in this study).

Our findings also highlight the disproportionate timing and location of contaminant delivery. For instance, 50% of TN, TP, and TSS loads occurred during just 36%, 19%, and 2% of monitored time, respectively, reflecting strong spatio-temporal variability in supply and transport. This pattern supports the HSHM concept^10^, which describes uneven biogeochemical processing and contaminant movement in time and space. At the sub-catchment scale, PKO-D contributed 62% of TSS loads but only 28% of flow, while a single six-day storm cluster accounted for 5% of annual TN, 14% of TP, and 32% of TSS loads across all sites. Such concentrated export events are difficult to capture with monthly sampling, reinforcing the value of high-frequency monitoring.

Comparison of ANN-derived load estimates with those based on monthly sampling revealed that ANN loads were 6%, 32%, and 85% greater for TN, TP, and TSS, respectively. These differences reflect the ability of high-frequency models to capture in-stream changes that occur between discrete samples. Similar discrepancies have been reported elsewhere, with many studies concluding that monthly sampling underestimates true loads and increases uncertainty^12,55–57^. For example, McDowell et al.^13^ showed that 48% of annual TN and 66% of annual TP loads in New Zealand occur during high flows; events that are often missed in monthly programmes. By capturing these dynamics, the method developed here improves understanding of the magnitude and likely sources of contaminant loads to the Waihi Estuary, providing a stronger foundation for setting achievable management targets.

Finally, our findings can inform catchment-scale modelling by enhancing the simulation of spatio-temporal nutrient fluxes. For example, ANN-derived contaminant signatures can be interpreted in relation to established biogeochemical concepts such as the four ‘control points’ described by Bernhardt et al.^9^ (permanent, activated, export, and transport). Yulianti et al.^58^ used a similar approach to improve SWAT+ model performance in Lake Ōkaro, calibrating streamflow sources and nitrogen transport dynamics using water and nitrogen isotopes. In our study, ANN outputs suggest that TN reaches the mainstem river in the Pongakawa sub-catchment predominantly via low-threshold export pathways consistent with vertical leaching and efficient shallow aquifer transport, resulting in persistently elevated baseflow concentrations. In contrast, the Pokopoko sub-catchment exhibited higher-threshold export, particularly for TP and TSS, where contaminants accumulated and were episodically delivered to the river during overland flow events.

### Translating results into management actions

Addressing the second objective of this study, our results show that ANN models can identify contaminant HSHM and resolve transport pathways at the catchment scale, while simultaneously capturing storm-event dynamics that drive load estimates. To inform management, these insights must be translated into actions that align with catchment-wide goals, in this case, achieving sustainable contaminant load targets for the Waihi Estuary. This process involves both prioritising and characterising model outputs.

Prioritisation is essential, as land managers must allocate limited resources where they will have the greatest impact. Sub-catchments with yields exceeding load targets per unit area are prime candidates for intervention, but gross contaminant export must also be considered to ensure meaningful reductions at the estuary scale. For example, Pongakawa and Pokopoko are high-priority areas for TN, TP, and TSS management due to both high yields and high total export. In contrast, Mangatoetoe, Puanene, and Wharere exceed yield thresholds but contribute relatively little gross export; improvements here would have limited influence on overall estuary loads and are therefore better suited for long-term rather than immediate action.

Characterisation of contaminant pathways ensures that management actions are tailored to the dominant transport processes within each sub-catchment. In Pongakawa and Pokopoko, elevated baseflow TN export indicates vertical leaching of NO₃ to groundwater as the key pathway. This is typical of intensively farmed New Zealand landscapes with porous volcanic or alluvial soils^59^. Here, soils act as export control points, accumulating nitrate until rainfall triggers leaching, while aquifers act as transport control points, conveying nitrate to streams. Because these pathways are largely controlled by inherent soil and geological properties, direct in-stream management is limited. The most effective strategies are those that reduce nitrogen accumulation and water movement at the source^60^. Recommended practices include limiting stocking rates, aligning fertiliser use with crop uptake and hydrology^61,62^, using organic nitrogen sources^63^, managing land-use sensitivities^62^, restricting tillage^60,61^, and modifying land surfaces to reduce permeability or slow overland flow^62^. Importantly, hydrological lag times mean that improvements may take years to be reflected in-stream; McDowell et al.^63^ estimate a median lag of 4.5 years for NO₃ in New Zealand, with detectable improvements often requiring up to a decade post-intervention.

Sub-catchment TP dynamics fall into two categories: baseflow-driven (e.g., PNG-M, PKO-UE, PKO-UW) and storm-event-driven (e.g., PKO-D, MGT, PUN, WHR-D). Elevated baseflow TP is largely attributable to the dissolution of P-rich volcanic geology^64^, which is difficult to manage without chemical interventions such as alum dosing^65^ which may not be feasible or desirable at catchment scales. Consequently, management is better directed toward storm-event-driven sub-catchments, where TP is predominantly particulate or sediment-bound. In these areas, contaminants are generally mobilised when rainfall generates sufficient overland flow, creating higher thresholds for export compared to nitrate in baseflow-dominated areas. Such dynamics present an opportunity for interceptive, co-management strategies that retain water and allow sediment to settle before reaching the river. Effective measures include retention bunds^66^, constructed wetlands^67^, protection of natural floodplains^68^, and riparian buffer strips^67,69^. At the same time, the most cost-effective way to address anthropogenic phosphorus is at its source, through fertiliser management^70^, identification of critical source areas^71^, and restriction of stock access^71^. Additional gains can be made by stabilising erosion-prone gully heads through prevention measures (e.g., topsoil reinforcement, vegetation barriers) or control methods (e.g., runoff diversion, gully reshaping, check dams, revegetation)^72^.

## Limitations and improvements

### Development and knowledge requirements

 A key limitation of this approach is the need for users to build and program each sensor station, which requires more technical expertise than most off-the-shelf (OTS) systems. However, these skills can be readily acquired through online resources designed to support community groups with little prior experience (Table S4). The main advantage of this method remains its cost: equipment costs are up to ten times lower than OTS systems, allowing wider spatial coverage across a catchment. In this study, the majority of equipment costs were associated with the CTD and nephelometer components, which accounted for approximately two-thirds of total station expenditure; alternative sensor configurations could further reduce costs where lower precision or different proxy measurements are acceptable. Additional requirements such as field technicians, autosamplers, and storm-event laboratory analyses meant that total programme costs were approximately 2.8 times higher than those of monthly sampling (Tables S5–S7). These expenses were primarily driven by event sample processing (64%), followed by sensor station equipment (30%), maintenance labour (4%), and autosampler deployment labour (2%). Despite higher overall costs, the added investment is minor when weighed against the risks of poorly informed land management actions or generic policies based on limited contaminant data.

### Model performance

Model performance was generally acceptable, though sites influenced by groundwater performed worse than mixed-flow sites. This limitation likely reflects the difficulty of detecting pulses of soluble contaminants from groundwater using the available sensors. Future improvements could involve deploying additional probes capable of capturing subtle groundwater signals, such as pH or redox sensors.

Paepae et al.^73^ propose accuracy targets for surrogate models, with R² values of 0.95–1.00 considered desirable, 0.90–0.95 acceptable, 0.85–0.90 tolerable, and 0.80–0.85 poor. By these standards, TN models in our study ranged from below poor to tolerable, TP from poor to acceptable, and TSS from poor to desirable. However, these thresholds were developed for commercial nitrate sensors, and Paepae et al. note that accuracy requirements depend on both environmental context and intended application. Given that our study used indirect sensor combinations to estimate concentrations, we argue that the spatio-temporal and hydrochemical insights gained offset the modest reduction in accuracy, particularly in applied contexts such as the Waihi Estuary, where outputs improve policy advice beyond what is possible with traditional monitoring.

### Uncertainty in discharge estimation

Discharge estimation represents a key source of uncertainty in this study and reflects a well-recognised challenge in New Zealand volcanic catchments. Streams within the Waihi Estuary catchment are predominantly soft-bottomed pumice systems that lack stable hydraulic controls, with highly mobile beds that complicate the development of reliable rating curves. These challenges were compounded by limited availability of spot flow measurements at several sites, necessitating the use of proxy or scaled flow relationships.

The objective of this study was not to achieve hydrological precision, but to resolve relative differences in contaminant export, timing, and dominant transport pathways across sub-catchments. Management interpretations are therefore robust to plausible discharge errors, as they are derived from consistent spatial and temporal patterns rather than absolute load magnitudes alone. This pragmatic approach is consistent with standard practice in many New Zealand catchment investigations, where resource constraints necessitate prioritisation of decision-relevant insights.

### Data coverage and representativeness

A further limitation relates to the size and frequency of datasets used to characterise hydrology and contaminant dynamics. Although ten monitoring sites improved spatial coverage, resource and equipment constraints limited storm-event sampling, discrete flow measurements, and the frequency of laboratory analyses, meaning ANN surrogate models are conditioned on the range of conditions observed during the study period.

Despite this, the combined datasets were sufficient to resolve consistent differences in contaminant behaviour between sub-catchments, particularly contrasts between baseflow-dominated and event-driven transport regimes. Management interpretations are therefore framed around relative patterns and dominant pathways rather than absolute load precision. Future studies could increase confidence by focusing on fewer strategically selected sites to improve storm-event coverage and discharge characterisation, reflecting the trade-off between spatial coverage and temporal resolution.

## Conclusions

This study demonstrates that traditional water quality monitoring can be substantially enhanced through the use of cost-effective DIY sensor stations combined with ANN models. High-frequency surrogate records captured hydrochemically significant storm events missed by monthly sampling, improving load estimates, particularly for TP and TSS.

Integrating model outputs within the HSHM framework provided several key benefits for water quality management. First, it identified sub-catchments and event timings where export and transport control points combined to create disproportionate contaminant risks. Second, it enabled a triage-based approach to management, directing immediate interventions to high-export sub-catchments while reserving lower-priority areas for long-term planning. Third, it supported efficient investment by showing that the majority of contaminant exports originated from a small number of sub-catchments, highlighting the value of targeted mitigation. Finally, the framework demonstrated the need for adaptive management, as shifting contaminant signatures under changing climatic and land-use conditions demand flexible prioritisation.

By coupling ANN modelling with the HSHM framework, this study presents a cost-effective and practical approach for disentangling complex contaminant transport dynamics. The approach provides more accurate and temporally resolved load estimates for sensitive estuarine environments such as the Waihi Estuary, while also generating actionable insights that support spatially explicit and tailored land management strategies. Although this framework requires greater resources than conventional monthly sampling, the improvements in accuracy, certainty, and management relevance justify the additional investment. Transferability to other agricultural catchments is likely where similar hydrological controls, sensor-constituent relationships, and calibration data availability exist.

## Supplementary Information

Below is the link to the electronic supplementary material.

Supplementary Material 1

## Data Availability

The data that support the findings of this study are available from the corresponding author upon reasonable request.
