# Supplementary material for: A do-it-yourself water quality sensor network to elucidate contaminant signatures and improve land management advice
Source: Sci Rep. 2026 Mar 16;16:11646. doi: 10.1038/s41598-026-43915-9 (PMC13062004; doi:10.1038/s41598-026-43915-9)
Supplement: Supplementary file 1 — Supplementary Material 1 [file 41598_2026_43915_MOESM1_ESM.docx]

### Supplementary Information

# A do-it-yourself water quality sensor network to elucidate contaminant signatures and improve land management advice

James E. Dare^1,2,3^, Deniz Özkundakci^1^, Richard W. McDowell^4,5^

^1^*School of Science, The University of Waikato, Hamilton, New Zealand.*

^2^*Bay of Plenty Regional Council, Tauranga, New Zealand.*

^3^*AquaWatch Solutions, Auckland, New Zealand.*

^4^*Faculty of Agriculture and Life Sciences, Lincoln University, Lincoln, New Zealand.*

^5^*AgResearch, Lincoln Science Centre, Lincoln, New Zealand.*

**Table S1** **Monitoring site codes and descriptions.** Short site codes are used throughout the manuscript to improve readability and consistency across figures and tables. The table provides the mapping between site codes, stream or sub-catchment, relative position within the catchment, and the original site names as used during field deployment and data collection by Bay of Plenty Regional Council.

| **Site Code** | **Stream** | **Relative Position** | **Original Site Name** |
| --- | --- | --- | --- |
| **MGT** | Mangatoetoe | Single site | Mangatoetoe at end of Black Rd |
| **PKO-UE** | Pokopoko | Upper East | Oeutehuehue at Farm Box Culvert |
| **PKO-UW** | Pokopoko | Upper West | Pokopoko at Allport Rd |
| **PKO-D** | Pokopoko | Downstream | Pokopoko at Old Coach Rd |
| **PNG-M** | Pongakawa | Mainstem | Pongakawa at Old Coach Rd |
| **PNG-TR1** | Pongakawa | Tributary 1 | Pongakawa trib at Rotoehu Rd |
| **PNG-TR2** | Pongakawa | Tributary 2 | Pongakawa Tributary at Old Coach Road |
| **PUN** | Puanene | Single site | Puanene at SH2 |
| **WHR-U** | Wharere | Upstream | Wharere at Maniatutu Rd |
| **WHR-D** | Wharere | Downstream | Wharere at Old Coach Rd |

**Table S2. A summary of ANN hyperparameter ranges across all retained site-parameter models.** Hidden (min), Hidden (med) and Hidden (max) indicate the minimum, median, and maximum number of hidden units in the single hidden layer. Epochs (min), Epochs (med), and Epochs (max) indicate the minimum, median, and maximum number of training epochs. Penalty (min), Penalty (med), and Penalty (max) indicate the range of L2 regularisation (weight decay) penalty values selected during tuning. LR (min), LR (med), and LR (max) indicate the range of learning rates selected. Only models meeting predefined performance criteria (NSE ≥ 0.5 and acceptable diagnostic behaviour) and used in subsequent load, yield, and baseflow analyses are included.

| **Parameter** | **n** | **Hidden (min)** | **Hidden (med)** | **Hidden (max)** | **Epochs (min)** | **Epochs (med)** | **Epochs (max)** | **Penalty (min)** | **Penalty (med)** | **Penalty (max)** | **LR (min)** | **LR (med)** | **LR (max)** |
| --- | --- | --- | --- | --- | --- | --- | --- | --- | --- | --- | --- | --- | --- |
| **TN** | 8 | 4 | 7.0 | 10 | 7 | 147 | 462 | 5.83E-10 | 7.67E-08 | 3.52E-03 | 0.00524 | 0.0810 | 0.250 |
| **TP** | 8 | 3 | 6.5 | 10 | 6 | 158 | 323 | 1.88E-10 | 1.11E-05 | 0.118 | 0.00119 | 0.0566 | 0.314 |
| **TSS** | 8 | 5 | 6.0 | 9 | 46 | 275 | 453 | 4.52E-10 | 0.0196 | 0.207 | 0.00785 | 0.124 | 0.282 |

**Table S3. Summary statistics for TN, TP, and TSS from the ten monitoring sites.** SD represents the standard deviation for each site, while the upper and lower quartiles are the 25th and 75th percentile values, respectively. All values aside from ‘n’ are in milligrams per litre (mg L^-1^). Statistics are calculated from routine samples only.

| **Parameter** | **Site Code** | **n** | **Median** | **SD** | **UQ** | **LQ** | **Min** | **Max** |
| --- | --- | --- | --- | --- | --- | --- | --- | --- |
| **TN** | MGT | 29 | 1.85 | 0.56 | 2.21 | 1.50 | 0.81 | 2.84 |
|  | PKO-UE | 24 | 1.79 | 0.28 | 1.97 | 1.74 | 1.24 | 2.42 |
|  | PKO-UW | 28 | 0.99 | 0.33 | 1.10 | 0.94 | 0.72 | 2.55 |
|  | PKO-D | 29 | 1.42 | 0.42 | 1.58 | 1.34 | 0.89 | 2.90 |
|  | PNG-M | 29 | 1.80 | 0.15 | 1.88 | 1.69 | 1.47 | 2.06 |
|  | PNG-TR1 | 29 | 2.03 | 0.31 | 2.31 | 1.89 | 1.61 | 2.74 |
|  | PNG-TR2 | 28 | 2.05 | 0.34 | 2.18 | 1.95 | 1.62 | 3.24 |
|  | PUN | 29 | 2.04 | 0.60 | 2.30 | 1.62 | 0.96 | 3.55 |
|  | WHR-U | 27 | 2.37 | 0.34 | 2.47 | 2.28 | 1.26 | 2.60 |
|  | WHR-D | 29 | 2.31 | 0.36 | 2.49 | 2.00 | 1.70 | 3.47 |
| **TP** | MGT | 29 | 0.103 | 0.094 | 0.116 | 0.089 | 0.057 | 0.493 |
|  | PKO-UE | 24 | 0.110 | 0.100 | 0.119 | 0.101 | 0.085 | 0.536 |
|  | PKO-UW | 28 | 0.091 | 0.085 | 0.105 | 0.084 | 0.070 | 0.516 |
|  | PKO-D | 29 | 0.114 | 0.144 | 0.133 | 0.094 | 0.071 | 0.832 |
|  | PNG-M | 29 | 0.114 | 0.028 | 0.131 | 0.104 | 0.091 | 0.215 |
|  | PNG-TR1 | 29 | 0.133 | 0.059 | 0.173 | 0.115 | 0.079 | 0.372 |
|  | PNG-TR2 | 28 | 0.127 | 0.073 | 0.149 | 0.116 | 0.102 | 0.487 |
|  | PUN | 29 | 0.133 | 1.222 | 0.155 | 0.120 | 0.091 | 6.727 |
|  | WHR-U | 27 | 0.119 | 0.048 | 0.125 | 0.114 | 0.105 | 0.338 |
|  | WHR-D | 29 | 0.126 | 0.122 | 0.136 | 0.115 | 0.100 | 0.764 |
| **TSS** | MGT | 29 | 19.0 | 69.7 | 30.0 | 13.3 | 2.1 | 340.0 |
|  | PKO-UE | 24 | 20.6 | 135.8 | 38.6 | 14.0 | 7.4 | 667.5 |
|  | PKO-UW | 28 | 55.9 | 358.6 | 110.1 | 35.8 | 10.1 | 1940.0 |
|  | PKO-D | 29 | 41.5 | 256.8 | 104.7 | 26.1 | 3.5 | 1413.3 |
|  | PNG-M | 29 | 19.7 | 42.9 | 67.8 | 10.0 | 2.0 | 145.5 |
|  | PNG-TR1 | 29 | 15.4 | 17.0 | 20.0 | 11.6 | 3.2 | 96.0 |
|  | PNG-TR2 | 28 | 10.5 | 125.5 | 19.4 | 5.8 | 1.7 | 672.0 |
|  | PUN | 29 | 20.6 | 70.5 | 35.8 | 14.0 | 3.6 | 392.0 |
|  | WHR-U | 27 | 3.8 | 21.7 | 6.8 | 2.4 | 0.9 | 89.0 |
|  | WHR-D | 29 | 16.2 | 78.8 | 29.1 | 12.0 | 5.9 | 440.0 |

**Table S4. Online resources to assist with the development of DIY monitoring stations.** All of these websites were used to research ideas. However, the final monitoring stations were based on content from EnviroDIY and the EnviroDIY Github page.

| **Resource** | **Description** | **Website** |
| --- | --- | --- |
| Stroud Water Research Center | A nonprofit organization dedicated to advancing the understanding and stewardship of freshwater systems through scientific research, education, and watershed restoration. The developers of EnviroDIY and Monitor My Watershed. | https://stroudcenter.org/ |
| EnviroDIY | An online community for do-it-yourself environmental science and monitoring. EnviroDIY is part of [WikiWatershed](https://wikiwatershed.org/), an initiative of [Stroud Water Research Center](https://stroudcenter.org/) designed to help people advance knowledge and stewardship of fresh water. | https://www.envirodiy.org/ |
| Monitor My Watershed | An online data sharing portal developed by the Stroud Water Research Institute. | https://monitormywatershed.org/ |
| EnviroDIY Github Page | A collection of code repositories to assist with DIY monitoring applications. | https://github.com/envirodiy |
| Hackaday | An engineering blog that includes articles on DIY water quality sensors. | https://hackaday.com/ |
| Yosemitech | Supplier of the Y11-A nephelometer used in this study, as well as numerous other water quality sensors. | https://www.yosemitech.com/ |
| METER Group | Supplier of the HYDROS-21 CTD sensor used in this study. | https://metergroup.com/ |
| DFRobot | DFRobot is a global provider of open-source hardware, robotics platforms, and STEM education kits, catering to DIY enthusiasts, educators, and industry developers | https://www.dfrobot.com/ |
| Hackster | An online community and platform for hardware developers, makers, and engineers to share projects, learn new skills, and collaborate on innovative technologies. | https://www.hackster.io/ |

**Table S5. Annual costs of the traditional monitoring network.** This includes all staff labour, sample analysis, and hydrological surveys associated with a traditional network design.

| **Description** | **Frequency** | **Cost per unit (NZD)** | **Unit** | **Units per sample run** | **Run cost (NZD)** | **Runs per year** | **Total Cost (NZD)** | **Comment** |
| --- | --- | --- | --- | --- | --- | --- | --- | --- |
| Staff labour | Monthly | $40 | Hour | 7.5 | $300 | 12 | $3,600.00 |  |
| Lab processing | Monthly | $100 | Sample | 10 | $1000 | 12 | $12,000.00 |  |
| Hydrological surveys | Annual | $45 | Hour | 40 | $1800 | 1 | $1,800.00 | It is assumed that it would take one week to gauge and process data for the nine sites that require flow data. These surveys would be carried out each year, and the full dataset would be used to develop a ‘ratio’ or relationship with a nearby hydrological site. |
| Total Annual Cost |  |  |  |  |  |  | $17,400.00 |  |

**Table S6. Costs associated with Mayfly sensor stations.** This includes all of the individual components required to create the ten sensor stations. GST stands for ‘Goods and Services Tax’ which is a New Zealand tax (15%) placed on the purchase of goods, including imported goods that are valued at over $1000 NZD.

| Item Description | Cost (NZD) | Quantity | Total (NZD) |
| --- | --- | --- | --- |
| Mayfly circuit board (pack 5) | $140 | 10 | $1,403.27 |
| Mayfly circuit board shipping | $145.20 | 1 | $231.09 |
| LTE modem | $133.83 | 10 | $1,338.26 |
| LTE adapter (Pack of 5) | $19.24 | 10 | $192.36 |
| HYDROS CTD sensor | $724.80 | 10 | $7,248.00 |
| HYDROS sensor shipping | $152.61 | 1 | $152.61 |
| HYDROS sensor import tax (GST) | $1,110.09 | 1 | $1,110.09 |
| Yosemitech nephelometer with brush | $1,019.60 | 10 | $10,196.00 |
| Yosemitech import tax (GST) | $1,529.48 | 1 | $1,529.48 |
| Cases | $30 | 10 | $300.00 |
| Misc Electrical | $10 | 10 | $100.00 |
| Misc Attachments | $50 | 10 | $500.00 |
| LiPO battery | $26.90 | 10 | $269.00 |
| Metal Stake | $10 | 20 | $200.00 |
| Solar Panel Equipment | $91.81 | 10 | $918.10 |
| Wingshield - Step up regulator | $10.36 | 10 | $103.61 |
| Wingshield - Step up regulator shipping | $55.47 | 1 | $55.47 |
| Wingshield - PCB | $13.87 | 1 | $13.87 |
| Wingshield - PCB Shipping | $22.20 | 1 | $22.20 |
| Wingshield - TTL to RS485 adapter (set 5) | $4.04 | 10 | $40.44 |
| Capacitors | $1 | 10 | $10.00 |
| Sum |  |  | $25,933.85 |
| Buffer (5%) |  |  | $1,296.69 |
| Total |  |  | $27,230.54 |
| Total per unit |  |  | $2,723.05 |

**Table S7. Cost of Mayfly network operation per year.** This table shows the additional costs associated with the Mayfly sensor network including: the initial capital cost, sensor maintenance, autosampler deployment, and autosampler lab processing. Costs are calculated for the Mayfly network and then added to traditional network cost to calculate the total cost.

| Description | Cost type | Frequency | Cost per unit (NZD) | Unit | Units per run | Run cost (NZD) | Runs per year | Cost per year | Comment |
| --- | --- | --- | --- | --- | --- | --- | --- | --- | --- |
| Autosampler deployment | Labour | Storm event | 40 | Hour | 2 | 80 | 8 | $640 | Assumed that eight events are captured across the network per year. Each event requires two hours labour to deploy and retrieve autosamplers |
| Autosampler Lab Processing | Contract | Storm event | 100 | Sample | 24 | 2400 | 8 | $19,200 | Current cost quoted by BOPRC lab per sample. |
| Routine Sensor Maintenance | Labour | Bi-monthly | 40 | Hour | 5 | 200 | 6 | $1,200 | Maintained every two months. It takes five hours to service the network. |
| Mayfly Equipment | Capital | One-off | $2,715 | Station | 10 | $27,150 |  | $9,050 | One off capital cost split over a three-year period. |
| Total Additional Mayfly |  |  |  |  |  |  |  | $30,090 | Additional cost of the enhanced Mayfly monitoring network. |
| Total Traditional Network |  |  |  |  |  |  |  | $17,400 | The cost of the status quo monitoring programme (Table S3) |
| Grand Total Mayfly Network |  |  |  |  |  |  |  | $47,490 | Total annual cost of the enhanced Mayfly monitoring network. |
